# Advancing precision: The role of digital workflows in modern implant dentistry

**DOI:** 10.6026/973206300212490

**Published:** 2025-08-31

**Authors:** Puneet Prasher, Anjali Tuteja, Falguni Amin, Resilda Sopaj, Diptansh Tuteja, Hasan Sharaf Eddin

**Affiliations:** 1 Department of Dental Surgery in Prosthodontics and Crown & Bridge, Sri Guru Ram Das Institute of Dental Sciences and Research, Punjab, India; 2 Department of Dental Surgery, Genesis Institute of Dental Sciences and Research, Ferozpur, Punjab, India; 3 Deparment of Dental Surgery, Karnavati School of Dentistry, Gandhinagar, Gujarat, India; 4 Integrated Second Level in Dentistry, Dental Doctor, University of Tirana, Faculty of Medicine, Tirana, Albania; 5 Department of Dental Surgery, Near East University, Northern Cyprus, Lefkoşa, Cyprus

**Keywords:** Digital workflow, CAD/CAM dentistry, Cone-beam computed tomography (CBCT), 3D printing in dentistry, Artificial intelligence (AI) in dentistry

## Abstract

Digital workflows have transformed implant dentistry by enhancing precision, predictability and patient outcomes through advanced imaging, computer-aided design/manufacturing and guided surgery. Therefore, it is of interest to review the key components of digital workflows, including 3D imaging, virtual treatment planning, dynamic navigation and CAD/CAM-fabricated prostheses. Clinical evidence demonstrates improved implant placement accuracy, reduced treatment times and enhanced aesthetics with digital integration. Challenges such as cost, learning curve and technology adoption are also discussed. Hence, there is a need for continued innovation and wider implementation to optimize implant dentistry practices.

## Background:

Over the last few decades, dentistry has undergone an evolution of advancements as new and improved technologies and techniques have changed how dental practices perform. Dentistry has made enormous progress in recent years and computerized applications have significantly contributed to the outcome, such as implant procedures. With intraoral scanners, dental lasers, computer-aided design, computer-aided manufacturing (CAD/CAM) and three-dimensional (3D) imaging, the modus operandi improves precision, efficiency and clinical effectiveness [1]. Digital workflow tools facilitate seamless communication among surgeons, restorative dentists and dental laboratories, thereby reducing uncertainty and enhancing the benefits of implant dentistry. With directed, navigated and occasionally robotically assisted surgical techniques that can be customized to each patient's specific medical, morphological and anatomical conditions, traditional methods of freehand implant positioning are giving way quickly [2]. By providing more accurate and effective methods than conventional analog techniques, digital implant workflows thrive in implant treatment planning. Among the many benefits of guided surgical intervention for implant placement is the instant restoration of both function and appearance with a temporary fix. The limitations of effective digital methods may still necessitate a hybrid process, which combines both digital and conventional approaches, even though digital technologies enhance accuracy and expedite dental implant procedures. Despite being technique-sensitive, computer-guided full-arch rehabilitation has undergone significant improvements thanks to advancements in 3D-manufactured surgical guides and open-source software [3]. Careful patient selection, thorough medical history evaluations and sophisticated imaging methods, such as cone beam computed tomography (CBCT), are all necessary for efficient preoperative planning. A variety of surgical guide types, including hands-on, dynamic, static, stackable and indistinguishable, may be used, depending on the complexity of the case; stackable guides provide increased workflow efficiency. To minimize complications through careful planning, postoperative management involves confirming that the prosthesis is correctly occluded and providing patients with clear aftercare instructions. It is anticipated that incorporating artificial intelligence into computerized processes will enhance implant planning and further inform design [4]. Therefore, it is of interest to describe digital workflows in modern implant dentistry.

## Digital imaging:

## Planning and treatment in implant dentistry:

Implantology in the digital era has evolved significantly. By leveraging cone-beam computed tomography (CBCT), intraoral scanning, facial scanning, artificial intelligence (AI) and dynamic navigation, digital workflows enable prosthetically driven implant placement, thereby reducing complications and optimizing outcomes.

## Digital imaging modalities:

Cone-beam computed tomography (CBCT) is central to implant planning, providing 3D visualization of bone and anatomical structures with submillimeter resolution (voxel sizes ranging from 0.076 to 0.4 mm) and lower radiation exposure compared to conventional computed tomography (CT) [5]. AI-enhanced CBCT provides automated nerve detection and bone density mapping (D1-D4), achieving 92.4% accuracy in predicting implant positions [6]. Intraoral scanners (*e.g.*, 3Shape TRIOS) capture soft tissue and dental surfaces with a precision of 7.9-39.6µm, surpassing the precision of traditional impressions [7]. Facial scanning (*e.g.*, Bellus3D) records smile dynamics at 24-60 frames per second, ensuring aesthetically driven designs [8].

## Digital treatment planning workflow:

Specialized software (*e.g.*, coDiagnostiX®) facilitates virtual implant positioning based on virtual tooth setups and safety zone mapping, reducing surgical time by 38% and improving accuracy by 72% compared to freehand methods [5]. AI tools provide bone quality assessment (with 89.7% agreement with histomorphometric analysis) and predictive modeling for implant success, thereby enhancing outcomes in complex cases with limited bone volume [6].

## Dynamic navigation and robotics:

Dynamic navigation systems offer real-time tracking with an accuracy of 0.3-0.5 mm, while robotic platforms (*e.g.*, Yomi) utilize haptic feedback for precise osteotomy preparation [9]. Augmented reality overlays enhance intraoperative decision-making, with navigated implants achieving 98.2% survival rates at 3 years, compared to 94.7% for conventional methods. These technologies perform well in challenging anatomies, reducing risks such as nerve damage.

## Clinical integration and challenges:

Reliable cross-platform data exchange via a standardized protocol is crucial in digital workflows. Validation, which includes checking guided surgery and CBCT scans, is performed to minimize errors [8]. High costs, steep learning curves and struggles with sharing data between proprietary systems are some of the challenges. For such a bottleneck, cloud-based data cybersecurity and formative training are necessary to be in place [10].

## Future outlook:

Potential Advances from New Technologies. New technologies are on the horizon that promise to bring improvements in the future. To predict outcomes, AI-driven analytics will incorporate patient-specific factors, such as bone turnover. In atrophic ridges manufactured using 3D printing, bioactive scaffolds will promote bone growth [9]. Reliance on fixed guides will decrease as augmented reality and cloud-based platforms enable handset design and real-time adjustments [10]. With the help of these advancements, digital workflows will become the norm, enabling accurate and patient-focused implant dentistry.

## Guided implant surgery using digital tools:

Guided implant surgery is a digitally assisted approach where prosthetic outcomes determine implant placement. Using CBCT and intraoral scans, a virtual plan is created and executed via a static guide, dynamic navigation, or robotic system, improving precision over freehand methods [11]. Static guides predetermine drill positioning, while dynamic navigation provides real-time on-screen guidance. Robotic systems, such as Yomi®, utilize a mechanical arm for highly controlled placement [12]. The digital workflow begins with CBCT (for bone anatomy) and intraoral scanning (for soft tissue and teeth). These datasets are merged in planning software (*e.g.*, Implant Studio®, Simplant®), where implants are virtually positioned for optimal prosthetic and anatomical fit. A 3D-printed surgical guide is then fabricated to ensure accurate drilling. Dynamic navigation bypasses the physical guide and instead tracks instruments in real time [11, 12]. Clinically, guided surgery enhances accuracy, with studies showing angular deviations of less than 5° and apex errors of ~1 mm, particularly near critical structures such as nerves or sinuses [13]. Flapless techniques, enabled by guided drilling, reduce tissue trauma, bleeding and the time required for healing [14]. Limitations include guide misfits due to soft tissue movement or scan mismatches. Static guides lack intraoperative adaptability and mucosa-supported guides may be unstable in edentulous cases. High costs for digital tools and training also limit accessibility [15, 16-17]. Future advancements include AI-driven planning for bone assessment and implant positioning, robotic systems with haptic feedback and augmented reality for collaborative planning [18, 19]. As technology evolves, guided surgery is poised to become the standard in implant dentistry.

## CAD/CAM fabrication of implant restoration:

CAD/CAM technologies enable the fabrication of implants with high accuracy by minimizing the number of production steps [20]. It starts with an accurate impression and involves three consecutive steps.

[1] Scanning

[2] Designing of the implant - CAD

[3] CAM production.

## Scanning:

Scanners capture the 3D geometry of dental structures, converting physical models into digital ones. They capture tooth preparation data, surrounding hard and soft tissues and occlusion by direct or indirect scanning.

## Direct scanning (Intraoral capture):

This method utilizes 3D optical systems to capture anatomy directly within the oral cavity. Some examples are laser scan, CEREC and Evolution 4D.

## Indirect scanning (Anatomical dental duplicate):

The capture of anatomical dental plaster casts typically employs a laser scanning method. Full-arch or large-area direct scanning exposes the patient to higher errors due to the stitching of an array of images [21].

## Designing of the implant - CAD:

After scanning and digitizing the components, the digital image files are imported into CAD software to design dental prostheses. Dental CAD/CAM systems typically use a specific file format and may operate as closed or open systems.

## Closed system:

Closed systems are ideal for streamlined workflows, such as chairside design and same-day restorations. For instance, a prefabricated titanium cylinder is scanned and a custom zirconia abutment is designed and milled to ensure an optimal fit. The zirconia abutment is then bonded to the titanium cylinder, eliminating the need for impressions and ensuring accuracy.

## Open system:

An open system may be preferred when more complex tasks are required (*e.g.*, in a commercial laboratory) [22]. A digital replica is created in the lab by scanning the tooth's impression, stone model, or die. The design is adapted to the opposing arch and the data is sent to a CAM center for milling the custom abutment.

## CAM production:

The CAM system enables the fabrication of accurate implant components, such as abutments and frameworks, in metal or ceramics, according to the 3D design in a CAD system [23]. The outer diameter (OD) of traditional molding can be removed by pulling, drilling, or grinding, resulting in distortion-free and porosity-free CAD-CAM systems. The end product is a long-lasting, high-quality custom implant part (made of titanium, Zirconia, or PEEK) and, by extension, longer-lasting restorations. Milling machines are classified based on their axis configuration.

3-axis devices

4-axis devices

5-axis devices

For larger types of frameworks and multi-implant cases, CAD/CAM technology offers the most significant advantage. CAD/CAM construction leads to improved patient comfort and reduces operating and clinical treatment times. Technical time and hands-on time between CAD/CAM and traditional lost-wax casting were reduced, allowing for additional work to be decreased, resulting in more efficient and precise outcomes [24, 25].

## Immediate loading and same-day restorations:

## Loading:

After implant surgery, dental implants are loaded instantly if a prosthesis or superstructure is attached within 72 hours or a week. Although retaining high implant yields, maintaining aesthetics and increasing patient comfort and satisfaction, this protocol shortens the duration of treatment and surgical procedures. An effective immediate loading process, which also typically leads to less bone loss over time, depends on primary implant stability. However, for long-term predictability, the method must be carefully planned and executed [26]. Through accurate implant placement, intraoperative impressions are not necessary and an immediate connection with a previously fabricated prosthesis is achieved. An attachable and removable guide system enables step-by-step treatment planning and performance of surgical and prosthetic treatment procedures, resulting in improved precision and reduced discomfort and operating time for the patient. With this procedure, patients can return to work and other regular routines, as function, appearance and sensation are quickly improved. Immediate loading in full-arch restorations has shown reasonable success rates, making it a reliable procedure that enables patients to start using their new teeth sooner rather than waiting months for the bone to heal [27].

## Restoration:

The two primary techniques used by CAD/CAM technology to shape dental restorations are additive and subtractive manufacturing. Manufacturing Subtraction: This procedure utilizes cutting tools guided by computer-generated paths to remove material within a prefabricated block. The prosthesis can be customized to meet specific needs by changing the external contour, width and finish harmony location and emergence profile. Using additive manufacturing (3D printing), dental devices are cost-effectively built layer by layer to form objects such as surgical guides, occlusal splints, dental models, custom impression trays and complete dentures [28, 29]. Chairside milling: Modern CAD-CAM technology has enabled dentists to manufacture their own crowns and implant abutments in a fraction of the time it used to take-a matter of hours. This allows us to perform a day of implant crown placement rather than working with the tedious handwork of days past [30]. Better cosmetic results and patient-specific solutions are made possible by its high degree of customization. Precise details are captured by digital impression techniques, which improve the marginal and inner alignment of restorations [31]. It is a hybrid restoration design composed of titanium that delivers implant-supported restorations with screw retention in a single visit.

## Full-arch implant restoration with digital transformation:

With digitization, immediate full-arch implant rehabilitation has found a new approach to practice, offering higher precision and patient acceptance. Prosthetic accuracy has increased thanks to advanced visualization (CBCT), intraoral scanning, computerized development, 3D printing and directed surgery, which has also decreased treatment time and discomfort. As an illustration of the effectiveness of digital workflows, a 70-year-old patient with low bone density was treated using an All-on-4 protocol with immediate loading [32].

## Problems and digital remedies:

The fit and functionality of the finished prosthesis may vary due to the multi-step nature of traditional full-arch rehabilitation. These days, digital tools improve reliability and interdisciplinary collaboration by streamlining the diagnosis, planning, surgery and prosthetics [33].

## Diagnostic phase:

By eliminating traditional impressions, intraoral scanners enhance accuracy and comfort. For optimal implant placement, CBCT provides a 3D assessment of the bone. Pre-treatment aesthetic previews are made possible by Digital Smile Design (DSD), which guarantees patient consent before procedure initiation [34]. Digital Therapy scheduling enables Prosthodontists, surgeons and laboratory technicians to communicate easily, reducing the need for appointments and boosting productivity [35].

## Phase of surgery:

## Guided implant positioning:

Surgical guides printed in 3D will ensure an accurate placement every time, particularly for immediate-loading procedures. Flawless techniques minimize trauma, making the outcome even more predictable and safe [36].

## In the prosthetic phase:

[1] Digital scans and permitted CBCT record the anatomical position very accurately.

[2] Using virtual implant planning to avoid critical structures (sinuses and nerves) was incorporated.

[3] Lastly, impromptu new provisional prostheses (fundamentally) were inserted, and guided surgery guarantees the accuracy of placement.

[4] Following osseointegration, the final prosthesis the titanium framework with a zirconia veneering is positioned [37].

It leads to high patient satisfaction, stable occlusion and superior prosthetic fit. Digital impressions contribute to time efficiency by reducing chairside time. Precision is enhanced through improved accuracy of prosthetic fabrication using digital tools. There is better collaboration between clinicians and technicians. Additionally, documentation quality and patient education are significantly improved [38]. Also a benefit of digital implant planning is the ability to try out different implant sizes and positions and quickly see the outcome in terms of screw-hole position and relationship to the restoration [39].

## Conclusion:

The management of complex full-arch dental rehabilitation has been greatly enhanced through the implementation of a digital workflow. Since its adoption, each case has been consistently addressed with precision, efficiency, and accuracy, leading to a marked improvement in patient satisfaction. As technology advances, clinicians are encouraged to adopt digital protocols as the standard of care in comprehensive dental treatment and to continuously stay abreast of the latest digital innovations.

## References

[1] Gawali N (2024). J Pharm Bioallied Sci..

[2] Blatz MB, Coachman C. (2023). Compend Contin Educ Dent..

[3] Abboud M (2020). Compend Contin Educ Dent..

[4] Odette J (2025). Oral Maxillofac Surg Clin North Am..

[5] Tahmaseb A (2018). Clin Oral Implants Res..

[6] Cui Z (2022). Nat Commun..

[7] Mangano F (2017). BMC Oral Health..

[8] Coachman C (2017). Int J Periodontics Restorative Dent..

[9] Block MS (2017). J Oral Maxillofac Surg..

[10] Joda T (2019). Comput Biol Med..

[11] Shi J (2023). Int J Implant Dent..

[12] Pettersson A (2014). J Prosthet Dent..

[13] Hultin M (2012). Clin Oral Implants Res..

[14] https://glidewelldental.com/.

[15] Marliére DA (2018). Eur J Dent..

[16] Iskandar M (2025). Oral Maxillofac Surg Clin North Am..

[17] Coachman C (2021). Int J Esthet Dent..

[18] Monaghesh E (2023). BMC Oral Health..

[19] Hölken F (2022). Medicina (Kaunas)..

[20] Abduo J, Lyons K. (2013). Int J Dent..

[21] Maiti N (2024). Bioinformation..

[22] Davidowitz G, Kotick PG. (2011). Dent Clin North Am..

[23] Watanabe H (2022). Dent Clin North Am..

[24] Cevik P (2022). Biomed Res Int..

[25] Fuster-Torres MA (2009). Med Oral Patol Oral Cir Bucal..

[26] Gamborena I (2021). J Esthet Restor Dent..

[27] Pardal-Peláez  B (2021). J Clin Exp Dent..

[28] Wittneben JG (2023). Clin Oral Implants Res..

[29] Beuer F (2008). Br Dent J..

[30] Atieh MA (2010). Int J Oral Maxillofac Implants..

[31] Blatz MB, Conejo J. (2019). Dent Clin North Am..

[32] Baroudi K, Ibraheem SN. (2015). J Int Oral Health..

[33] D'Arienzo LF (2020). BioCensus..

[34] Papaspyridakos P (2012). Int J Oral Maxillofac Implants..

[35] Wulfman C (2020). J Prosthet Dent..

[36] Moraschini V, Barboza EP. (2016). Int J Oral Maxillofac Surg..

[37] Chao D (2020). J Prosthet Dent..

[38] Chen J (2019). J Prosthet Dent..

[39] Rahman RN (2022). J Calif Dent Assoc..

